# Low and exacerbated levels of 1,5-anhydroglucitol are associated with cardiovascular events in patients after first-time elective percutaneous coronary intervention

**DOI:** 10.1186/s12933-016-0459-5

**Published:** 2016-10-11

**Authors:** Shuhei Takahashi, Kazunori Shimada, Katsumi Miyauchi, Tetsuro Miyazaki, Eiryu Sai, Manabu Ogita, Shuta Tsuboi, Hiroshi Tamura, Shinya Okazaki, Tomoyuki Shiozawa, Shohei Ouchi, Tatsuro Aikawa, Tomoyasu Kadoguchi, Hamad Al Shahi, Takuma Yoshihara, Makoto Hiki, Kikuo Isoda, Hiroyuki Daida

**Affiliations:** Department of Cardiovascular Medicine, Juntendo University Graduate School of Medicine, 2-1-1, Hongo, Bunkyo-ku, Tokyo, 113-8421 Japan

**Keywords:** Postprandial hyperglycemia, 1,5-Anhydroglucitol, Coronary artery disease, Cardiovascular events

## Abstract

**Background:**

Postprandial hyperglycemia plays an important role in the pathogenesis of coronary artery disease and cardiovascular events. Serum 1,5-anhydroglucitol (1,5-AG) levels are known to be a clinical marker of postprandial hyperglycemia. However, the impact of 1,5-AG level on cardiovascular events has not been fully investigated.

**Methods:**

We enrolled 240 consecutive patients who had undergone first-time elective percutaneous coronary intervention (PCI) with follow-up angiography within 1 year. We excluded patients with a history of acute coronary syndrome, advanced chronic kidney disease (estimated glomerular filtration rate <30 mL/min/1.73 m^2^), or uncontrolled diabetes mellitus (HbA1c ≥7.0 %). Fasting blood glucose (FBS), HbA1c, and 1,5-AG levels were measured prior to PCI and at the time of follow-up angiography. Clinical events, including target lesion revascularization, target vessel revascularization, and revascularization of new lesions, were evaluated.

**Results:**

Subjects were divided into two groups according to clinical outcomes: the Event (+) group (n = 40) and the Event (−) group (n = 200). No significant differences were observed, except for the number of diseased vessels and the prevalence of statin use, in baseline clinical characteristics between the two groups. Serum levels of 1,5-AG at follow-up were significantly lower in the Event (+) group than in the Event (−) group (P = 0.02). A significant reduction in 1,5-AG level from baseline to follow-up was observed in the Event (+) group compared with the Event (−) group (P = 0.04). The association between 1,5-AG levels at follow-up and clinical events remained significant after adjustment for independent variables, including FBS and HbA1c levels (P = 0.04).

**Conclusions:**

Low and exacerbated levels of 1,5-AG were associated with cardiovascular events in the present study, indicating that postprandial hyperglycemia is an important risk factor for adverse clinical events even in patients with HbA1c < 7.0 %, following first-time elective PCI.

## Background

Postprandial hyperglycemia is considered to be a risk factor for atherosclerotic diseases such as coronary artery disease (CAD) [1–5]. Serum 1,5-anhydroglucitol (1,5-AG) levels better reflect short-term glucose control and postprandial hyperglycemia than do hemoglobin A1c (HbA1c) levels [6, 7]. Therefore, the measurement of 1,5-AG levels may have utility as a clinical marker of glycemic control in patients with CAD. Indeed, previous studies have reported that 1,5-AG levels are associated with vascular endothelial dysfunction [8], carotid atherosclerosis [9], and CAD [10, 11]. In addition, 1,5-AG levels predicted cardiovascular events in two previous population-based cohorts and a clinical study with relatively small-sample sizes [12–14].

However, the association between 1,5-AG levels and cardiovascular events in patients with CAD is unclear. Moreover, the American Diabetes Association and the European Association for the Study of Diabetes have recommended that a reasonable HbA1c goal for adult patients with diabetes mellitus (DM) is <7.0 % (53 mmol/mol) [15, 16]. To date, appropriate glycemic control markers for preventing cardiovascular events in patients with CAD and HbA1c level <7.0 % have yet to be reported. Therefore, we examined whether serum 1,5-AG levels can predict adverse cardiovascular events in patients with HbA1c level <7.0 % after first-time elective percutaneous coronary intervention (PCI).

## Methods

### Study subjects

The present study was a retrospective observational study. The study was approved by the institutional review board at the Juntendo University and performed in accordance with the principals of the Declaration of Helsinki. Written informed consent was obtained from all patients. First, we recruited 538 consecutive subjects after first-time elective PCI with drug-eluting stents (DES) between April 2011 and January 2015. Patients meeting the following criteria were excluded: no follow-up angiography, a history of acute coronary syndrome (ACS), estimated glomerular filtration rate <30 mL/min/1.73 m^2^ calculated using the modification of diet in renal disease equation modified with a Japanese coefficient using baseline serum creatinine [17], HbA1c ≥7.0 %, under treatment with sodium glucose co-transporter 2 inhibitors, a history of gastrectomy, or previous bare-metal stent implantation.

### Data collection

Clinical characteristics, including age, gender, body-mass index (BMI), smoking habit, a history of hypertension, history of DM, family history of CAD, ejection fraction, blood pressure, and concomitant use of medication, were collected at the time of PCI. Biochemical variables from blood samples were evaluated after an overnight fast. Hypertension was defined as a systolic blood pressure ≥140 mmHg, a diastolic blood pressure ≥90 mmHg or treatment with antihypertensive medication. DM was defined as a fasting blood glucose ≥126 mg/dL, serum HbA1c level ≥6.5 %, or treatment with an oral antihyperglycemic drug or insulin injections. Current smokers were defined having smoked at the time of PCI or had quit within 1 year prior to the study period. We defined ACS as unstable angina pectoris (UAP), non-ST segment elevation myocardial infarction (NSTEMI), or ST segment elevation myocardial infarction (STEMI). UAP was defined as having angina at rest or in an accelerating pattern with negative cardiac biomarkers and transient ischemic ST segment shift. Myocardial infarction was defined as an increase in serum cardiac enzymes (troponin, CK ≥ twofold increase).

### Blood samples

We obtained blood samples immediately prior to coronary stenting and at the time of follow-up angiography within 1 year. Samples were stored at −80 °C for later measurement of 1,5-AG levels. Serum levels of 1,5-AG were measured by a colorimetric method (Nippon Kayaku, Tokyo, Japan) using a Lana 1,5-AG auto liquid automatic analyzer (JCA-BM 8060, JEOL Ltd., Tokyo, Japan). HbA1c levels were measured by high-performance liquid chromatography (TOHSOH, Tokyo, Japan). Serum levels of total cholesterol, triglycerides and high-density lipoprotein (HDL) cholesterol were measured by standard enzymatic methods (Kainos, Tokyo, Japan), and low-density lipoprotein (LDL) cholesterol values were measured by the direct method. High-sensitivity C-reactive protein (CRP) levels were measured by latex photometric immunoassay (CRP-Latex “Seiken,” Denka Seiken, Inc.) with an autoanalyzer (Hitachi H7350). Blood glucose levels were measured using an enzymatic method. These assays have inter- and intra-assay coefficients of variation of 0.5 and 0.4 % for 1,5-AG, 1.1 and 0.9 % for HbA1c, 1.9 and 0.5 % for blood glucose, 1.4 and 1.5 % for total cholesterol, 1.3 and 0.6 % for triglycerides, 2.1 and 1.1 % for HDL-cholesterol, and 1.9 and 0.6 % for LDL-cholesterol, respectively.

### Angiographical analysis

Coronary angiography was performed on all patients at baseline. The extent of stenosis was measured by the number of stenotic vessels recorded as 1-, 2-, 3-vessel disease or stenosis of the left main artery. Narrowing of the pre-stenotic diameter of the blood vessels 75 % was considered significant stenosis. Quantitative coronary angiography (QCA) assessments were performed in all subjects. All QCA analyses were performed by a technician without any knowledge of the study results as previously described [18, 19]. Absolute values for the mean reference diameter and minimal luminal diameter were determined. All PCI procedural decisions, including device selection and adjunctive pharmacotherapy, were made at the discretion of the individual PCI operator. Intravenous unfractionated heparin and intracoronary nitroglycerin were administered prior to PCI. After DES implantation, angiographic optimization was performed using high-pressure dilatation to achieve an acceptable angiographic result. Intravascular ultrasound was performed at the operator’s discretion. Procedural success was defined as a residual stenosis <20 % without major complications. Dual antiplatelet therapy (100 mg aspirin with 200 mg ticlopidine or 75 mg clopidogrel) was prescribed to all patients treated with DES until follow-up coronary angiography at the earliest. Target lesion revascularization (TLR) was defined as repeat PCI within the index procedure stent or 5 mm edge. Target vessel revascularization (TVR) was defined as repeat PCI in the target vessel. A new lesion was defined as de novo lesions requiring revascularization except for TLR and TVR.

### Statistical analysis

All results are expressed as percentages for categorical variables and as the mean ± standard deviation for continuous variables. Baseline data were compared using Student’s *t* test or the Mann–Whitney *U* test for continuous variables. Categorical variables were compared using the Chi square test. Multivariate logistic regression analysis was performed to identify independent factors for adverse clinical events, including age, gender, BMI, and all variables with a P value <0.2 on univariate logistic regression analysis. P values <0.05 were considered statistically significant. All data were analyzed using JMP version 11.0 for Windows (SAS Institute, Cary, NC, USA).

## Results

### Patient characteristics

**Fig. 1:**
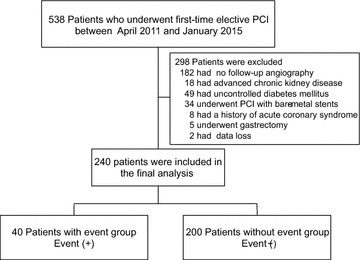
Flow chart of study population. *PCI* percutaneous coronary intervention

During the study period, 538 patients underwent first-time elective PCI in our institution. We excluded 182 patients without follow-up angiography, 18 patients with advanced chronic kidney disease, 49 patients with HbA1c ≥7.0 % at baseline, 34 patients who underwent PCI with bare-metal stents, 8 patients with ACS, 5 patients with a history of gastrectomy, and 2 patients with data loss. As a result, a total of 240 consecutive patients who had undergone first-time elective PCI were enrolled into the present study (Fig. 1). The mean follow-up period was 252 days (range 105–360 days). During the follow-up, 13 patients received TLR, 4 patients received TVR, and 23 patients received revascularization for de novo lesions.

Subjects were divided into two groups according to clinical outcomes: the Event (+) group (n = 40) and the Event (−) group (n = 200). Clinical characteristics, laboratory findings, and concomitant use of medications are shown Tables 1, 2 and 3, respectively. At baseline, the number of diseased vessels significantly differed between the two groups. The prevalence of statin use was significantly higher in the Event (−) group at baseline. No significant differences in clinical characteristics were observed between the two groups at follow-up.

**Table 1:** Patient characteristics at baseline

	Event (+) (n = 40)	Event (−) (n = 200)	P value
Age (years)	68 ± 9	69 ± 10	0.54
Male, n (%)	32 (80)	164 (82)	0.77
BMI (kg/m^2)^	24.6 ± 3.1	24.4 ± 3.2	0.74
Hypertension, n (%)	30 (75)	148 (74)	0.89
Diabetes mellitus, n (%)	13 (33)	57 (29)	0.61
Current smoker, n (%)	10 (25)	38 (19)	0.40
Family history of premature CAD, n (%)	6 (15)	52 (26)	0.11
EF (%)	62 ± 12	66 ± 8	0.10
No. of diseased vessels			0.03
One, n (%)	9 (22)	93 (46)	
Two, n (%)	18 (45)	73 (37)	
Three, n (%)	12 (30)	32 (16)	
Stenosis of left main artery, n (%)	1 (3)	2 (1)	

Data are presented as mean ± SD or number (%)

*BMI* body-mass index, *CAD* coronary artery disease, *EF* ejection fraction

**Table 2:** Clinical and laboratory findings at baseline and follow-up

	Baseline			Follow-up		
	Event (+) (n = 40)	Event (−) (n = 200)	P value	Event (+) (n = 40)	Event (−) (n = 200)	P value
Systolic blood pressure (mmHg)	148 ± 26	143 ± 21	0.20	147 ± 25	142 ± 23	0.22
Diastolic blood pressure (mmHg)	82 ± 14	79 ± 13	0.15	82 ± 13	79 ± 14	0.23
HDL cholesterol (mg/dl)	45 ± 10	44 ± 12	0.75	49 ± 14	46 ± 13	0.19
LDL cholesterol (mg/dl)	96 ± 30	90 ± 23	0.17	84 ± 25	84 ± 20	0.98
Triglyceride (mg/dl)	137 ± 55	127 ± 61	0.34	124 ± 86	119 ± 57	0.66
Creatinine (mg/dl)	0.79 ± 0.17	0.79 ± 0.20	0.82	0.83 ± 0.21	0.80 ± 0.21	0.36
Hs-CRP (mg/L), median (25th–75th)	0.08 (0.04–0.15)	0.08 (0.03–0.22)	0.75	0.05 (0.03–0.13)	0.07 (0.03–0.17)	0.65
BNP (pg/ml), median (25th–75th)	34 (23–62)	30 (14–68)	0.28	28 (14–77)	30 (15–56)	0.90

Data are presented as mean ± SD

*HDL* high-density lipoprotein, *LDL* low-density lipoprotein, *CRP* C-reactive protein, *BNP* brain natriuretic peptide

**Table 3:** Medical therapy at baseline and follow-up

	Baseline			Follow-up		
	Event (+) (n = 40)	Event (−) (n = 200)	P value	Event (+) (n = 40)	Event (−) (n = 200)	P value
Aspirin, n (%)	39 (98)	199 (99)	0.28	40 (100)	199 (99)	0.55
Calcium-channel blocker, n (%)	19 (48)	103 (52)	0.64	20 (50)	110 (55)	0.56
Beta-blocker, n (%)	18 (45)	104 (52)	0.42	15 (38)	104 (52)	0.09
ACE inhibitor or ARB, n (%)	19 (48)	104 (52)	0.60	21 (53)	109 (55)	0.82
Statin, n (%)	20 (50)	137 (69)	0.03	34 (85)	175 (88)	0.67
Sulfonylurea, n (%)	4 (10)	13 (7)	0.45	4 (10)	13 (7)	0.45
DPP-4 inhibitor, n (%)	3 (8)	24 (12)	0.39	4 (10)	24 (12)	0.71
Metformin, n (%)	3 (8)	12 (6)	0.73	3 (8)	12 (6)	0.73
Pioglitazone, n (%)	1 (3)	5 (3)	1.00	1 (3)	5 (3)	1.00
Glinide, n (%)	2 (5)	7 (4)	0.66	2 (5)	6 (3)	0.54
Alpha-glucosidase inhibitor, n (%)	5 (13)	11 (6)	0.14	4 (10)	9 (5)	0.20
Insulin, n (%)	4 (10)	7 (4)	0.11	4 (10)	7 (4)	0.11

Data are presented as number (%)

*ACE* angiotensin converting enzyme, *ARB* angiotensin receptor blocker, *DPP-4* dipeptidyl peptidase-4

### Change in glycemic markers

**Fig. 2:**
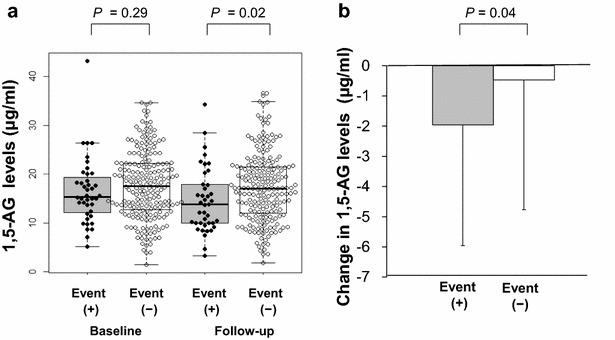
Serum 1,5-AG levels and change in 1,5-AG levels. **a** Serum levels of 1,5-AG at baseline and follow-up in the two groups. Data are presented as a *box plot* with the 25th–75th percentiles containing the *median line* and *lines* depicting the 10th–90th percentiles. 1,5-AG, 1,5-anhydroglucitol. **b** Changes in 1,5-AG levels between the two groups. Data are presented as mean ± SD

Regarding baseline glycemic markers, no significant differences in FBS, HbA1c, or 1,5-AG levels were observed between the two groups. However, at follow-up, serum levels of 1,5-AG (14.5 ± 6.6 vs. 17.3 ± 7.0 μg/mL, P = 0.02), but not FBS (100 ± 19 vs. 97 ± 18 mg/dl, P = 0.31) or HbA1c (6.0 ± 0.7 vs 5.9 ± 0.5 %, P = 0.84), were significantly different between the Event (+) group and the Event (−) group (Table 4). Interestingly, a significant reduction in 1,5-AG levels from baseline was observed in the Event (+) group compared to the Event (−) group (−2.0 ± 4.0 vs. −0.5 ± 4.2 μg/mL, P = 0.04) at follow-up (Fig. 2).

**Table 4:** Glycemic markers at baseline and follow-up

	Baseline			Follow-up		
	Event (+) (n = 40)	Event (−) (n = 200)	P value	Event (+) (n = 40)	Event (−) (n = 200)	P value
FBS (mg/dl)	100 ± 15	97 ± 19	0.41	100 ± 19	97 ± 18	0.31
HbA1c (%)	5.9 ± 0.5	5.9 ± 0.5	0.77	6.0 ± 0.7	5.9 ± 0.5	0.84
1,5-AG (μg/ml)	16.5 ± 6.7	17.7 ± 7.1	0.29	14.5 ± 6.6	17.3 ± 7.0	0.02

Data are presented as mean ± SD

*FBS* fasting blood glucose, *HbA1c* hemoglobin A1C, *1,5-AG* 1,5-anhydroglucitol

The association between 1,5-AG level and clinical adverse events remained significant after adjustment for other variables, including age, male gender, BMI, family history of premature CAD, HDL-cholesterol, statin therapy, and multiple diseased vessels (OR 0.94; 95 % CI 0.89–0.99; P = 0.04; Table 5).

**Table 5:** Results of univariate and multivariate analyses at follow-up

	Univariate			Multivariate		
	OR	95 % CI	P value	OR	95 % CI	P value
Age (years)	0.99	0.95–1.02	0.52	0.96	0.92–1.00	0.05
Male—yes	0.88	0.39–2.19	0.77	1.06	0.41–2.95	0.91
BMI (kg/m^2^)	0.98	0.87–1.09	0.73	0.98	0.86–1.11	0.72
Current smoker—yes	1.42	0.61–3.08	0.40			
Family history of premature CAD—yes	0.50	0.18–1.17	0.11	0.37	0.12–0.96	0.04
Systolic blood pressure (mmHg)	1.01	0.99–1.03	0.21			
Diastolic blood pressure (mmHg)	1.02	0.99–1.04	0.23			
HDL cholesterol (mg/dL)	1.02	0.99–1.04	0.19	1.02	0.99–1.05	0.12
LDL cholesterol (mg/dL)	1.00	0.98–1.02	0.98			
Triglyceride (mg/dL)	1.00	0.99–1.01	0.66			
Creatinine (mg/dL)	2.08	0.41–9.63	0.36			
Hs-CRP (mg/L)	0.79	0.28–1.31	0.46			
FBS (mg/dL)	1.01	0.99–1.03	0.33			
HbA1c (%)	0.94	0.49–1.70	0.84			
1,5-AG (μg/mL)	0.94	0.89–0.99	0.02	0.94	0.89–0.99	0.04
Hypoglycemic therapy—yes	1.47	0.66–3.12	0.34			
Anti-hypertensive therapy—yes	0.96	0.39–2.72	0.94			
Statin therapy—yes	0.46	0.23–0.92	0.03	0.44	0.21–0.93	0.03
Multiple diseased vessels—yes	2.99	1.41–6.98	0.004	3.41	1.54–8.23	0.002

Age, male gender, BMI, and variables with a P value <0.20 on Univariate analysis were analyzed by multivariate logistic regression

*OR* odds ratio, *CI* confidence interval, *BMI* body-mass index, *HDL* high-density lipoprotein, *LDL* low-density lipoprotein, *FBS* fasting blood glucose, *HbA1c* hemoglobin A1C, *1,5-AG* 1,5-anhydroglucitol

## Discussion

The results of the present study indicate low and exacerbated levels of 1,5-AG are associated with clinical events in patients with first-time elective PCI. Previous reports demonstrating the association between 1,5-AG and cardiovascular complications have included in population-based cohort studies [12, 13] and patients with CAD, including those with relatively high HbA1c levels [14]. This is the first study, to report serum 1,5-AG levels as significantly associated with clinical events in CAD patients with HbA1c levels <7.0 %.

DM and impaired glucose tolerance play an important role in the pathogenesis of cardiovascular events, including the incidence of restenosis following PCI [20]. Nonetheless, target values of glycemic control in patients with DM have not been fully determined. Previous clinical trials have demonstrated little benefit of intensive glycemic therapy to lower HbA1c levels in patients with DM for the prevention of cardiovascular events [21–23]. In addition, the utility of other glycemic markers as clinical predictors of adverse cardiovascular events in patients with CAD remains unknown. Recently, postprandial hyperglycemia has been posited as a risk factor for CAD [1–5, 24]. We previously reported that patients with multivessel CAD had slightly but significantly higher blood glucose levels at 1 h after a 75-g oral glucose tolerance test compared to subjects without CAD, even in subjects with normal glucose tolerance [25]. Therefore, there is a clinical need for a glycemic marker that reflects postprandial hyperglycemia in order to identify patients at high risk of cardiovascular events despite the absence of DM.

Serum 1,5-AG levels better reflect short-term glucose control and postprandial hyperglycemia than hemoglobin A1c (HbA1c) levels [6, 7]. Previous studies have reported that 1,5-AG levels are associated with vascular endothelial dysfunction [8] and cardiovascular diseases [9–11]. In addition, 1,5-AG levels were found to predict cardiovascular events in two population-based cohorts and a clinical study with a small-sample size [12–14]. Watanabe et al. reported that the adjusted hazard ratios (HRs) of all cardiovascular diseases in men increased linearly (P = 0.004) and the adjusted HR was 2.22 (95 % CI 1.24–3.98) in the lowest 1,5-AG category in a population-based cohort study comprising 2095 Japanese individuals without a history of CAD or stroke [12]. In the Atherosclerosis Risk in Communities study involving 11,106 participants without cardiovascular disease at baseline, Selvin et al. recently showed that subjects with DM and 1,5-AG <6.0 mg/mL had an increased risk of CAD (HR 3.85, 95 % CI 3.11–4.78), stroke (HR 3.48, 95 % CI 2.66–4.55), heart failure (HR 3.50, 95 % CI 2.93–4.17), and death (HR 2.44, 95 % CI 2.11–2.83) compared with subjects with 1,5-AG **≥**6 mg/mL and no history of DM [13]. In 141 patients after PCI, Fujiwara et al. [14] demonstrated that 1,5-AG levels were significantly lower in patients with any coronary revascularization (P = 0.005) and target lesion revascularization. Moreover, appropriate glycemic control markers for preventing cardiovascular events in patients with CAD and HbA1c levels <7.0 % have yet to be reported. In the present study, low and exacerbated levels of 1,5-AG were important risk factors for adverse clinical events in patients with HbA1c levels <7.0 % after first-time elective PCI. Therefore, the measurement of 1,5-AG level may be important, not only for the assessment of postprandial hyperglycemia, but identifying patients at high risk of adverse clinical events, even in CAD patients with HbA1c levels <7.0 %.

Several recent studies have reported the associations between 1,5-AG levels and cardiovascular disorders [26–29]. A low 1,5-AG levels has been associated with elevated cardiac troponin T and prospectively associated with the 6-year incident elevation in troponin T levels in patients with DM [26]. This may be linked to a possible harmful effect of hyperglycemic peaks on the myocardium or on the microvascular supply to the myocardium [26]. However, whether the associations between a low 1,5-AG levels and myocardial damage are independent of average glucose level remains unclear [26]. A recent study demonstrated that the 1,5-AG level is negatively associated with hypoglycemia in patients with well-controlled DM receiving insulin therapy [27]. This finding suggests that low 1,5-AG levels are associated with severe glycemic variability due to postprandial hyperglycemia and enhanced hypoglycemia, which affect the 1,5-AG level in opposing directions, in patients with well-controlled DM with relatively low HbA1c levels. In contrast, glycated albumin levels have been found to be more closely correlated with CAD than 1,5-AG and HbA1c levels in 272 Chinese subjects [28]. Compared with 1,5-AG levels, glycated albumin and HbA1c levels have been found to provide superior discrimination for carotid wall thickness in community-dwelling Japanese subjects with glucose intolerance [29]. Both these studies had a cross-sectional design. In the former study assessed by the coronary stenosis index, the clinical characteristics, including age, gender, lipid profile, prevalence of DM, and concomitant use of medications for dyslipidemia and DM, were significantly different. Therefore, several confounding factors may have affected the results. Further prospective studies in a larger population are required to assess the relationship between the 1,5-AG level and cardiovascular diseases.

The present study has several limitations. First, this was a single-center study and the study sample size was small. Second, subjects were exclusively patients who had undergone first-time elective PCI and follow-up angiography. Third, we were unable to determine all clinical characteristics with an effect on 1,5-AG levels (e.g., renal glycosuria). Forth, there was individual variation in the follow-up angiography period. Fifth, we did not perform a 75gOGTT in all non-diagnosed DM patients in the present study. This may have led to underestimation of the number of patients with DM. We think that the measurement of serum levels of 1,5-AG may be useful for evaluating not only postprandial hyperglycemia but also DM in the clinical setting without a 75gOGTT, even in those with HbA1c <7.0 %. Finally, the study follow-up duration was not long term. Accordingly, it was relatively difficult to evaluate major adverse cardiovascular events, including all-cause mortality, nonfatal myocardial infarction, and nonfatal stroke. Therefore, the results of the present study require further confirmation in a larger population cohort over a long-term period.

## Conclusions

The present study demonstrated that low and exacerbated levels of 1,5-AG were associated with cardiovascular events, indicating postprandial hyperglycemia is an important risk factor for adverse clinical events even in patients with HbA1c levels <7.0 %, after first-time elective PCI.

## Abbreviations

CAD: coronary artery disease
1,5-AG: 1,5-anhydroglucitol
HbA1c: hemoglobin A1c
DM: diabetes mellitus
PCI: percutaneous coronary intervention
DES: drug-eluting stents
ACS: acute coronary syndrome
BMI: body-mass index
UAP: unstable angina pectoris
NSTEMI: non-ST segment elevation myocardial infarction
STEMI: ST segment elevation myocardial infarction
HDL: high-density lipoprotein
LDL: low-density lipoprotein
CRP: C-reactive protein
QCA: quantitative coronary angiography
TLR: target lesion revascularization
TVR: target vessel revascularization
